# Hexane Fraction of *Adenophora triphylla* var. *japonica* Root Extract Inhibits Angiogenesis and Endothelial Cell-Induced Erlotinib Resistance in Lung Cancer Cells

**DOI:** 10.3390/molecules29030597

**Published:** 2024-01-25

**Authors:** Hyun-Ji Park, Jae-Hoon Jeong, Yung-Hyun Choi, Shin-Hyung Park

**Affiliations:** 1 Department of Pathology, College of Korean Medicine, Dong-eui University, Busan 47227, Republic of Korea; 14554@deu.ac.kr (H.-J.P.); 15224@deu.ac.kr (J.-H.J.); 2 Department of Biochemistry, College of Korean Medicine, Dong-eui University, Busan 47227, Republic of Korea; choiyh@deu.ac.kr

**Keywords:** *Adenophora triphylla* var. *japonica*, lung cancer, angiogenesis, erlotinib resistance, VEGFR2

## Abstract

The aim of this study was to investigate the anti-angiogenic effects of the hexane fraction of *Adenophora triphylla* var. *japonica* root extract (HAT) and its influence on the development of erlotinib resistance in human lung cancer cells. HAT significantly reduced the migration, invasion, and tube formation of human umbilical vein endothelial cells (HUVECs). The phosphorylation levels of vascular endothelial growth factor receptor 2 (VEGFR2) and its downstream molecules were decreased via HAT, indicating its anti-angiogenic potential in endothelial cells (ECs). A docking analysis demonstrated that β-sitosterol and lupeol, representative components of HAT, exhibit a high affinity for binding to VEGFR2. In addition, conditioned media from HAT-pretreated H1299 human lung cancer cells attenuated cancer-cell-induced chemotaxis of HUVECs, which was attributed to the decreased expression of angiogenic and chemotactic factors in H1299 cells. Interestingly, co-culture of erlotinib-sensitive PC9 human lung cancer cells with HUVECs induced erlotinib resistance in PC9 cells. However, co-culture with HAT-pretreated HUVECs partially restored the sensitivity of PC9 cells to erlotinib. HAT inhibited the development of erlotinib resistance by attenuating hepatocyte growth factor (HGF) production by ECs. Taken together, our results demonstrate that HAT exerts its anticancer effects by regulating the crosstalk between ECs and lung cancer cells.

## 1. Introduction

Lung cancer is a leading cause of cancer death, accounting for approximately 18% of all cancer deaths worldwide. It is the most common cancer in men and the third most common cancer in women globally [1]. The incidence of lung cancer is closely linked to smoking, exposure to air pollution, and inhalation of other carcinogens such as asbestos [1]. Despite improvements in the prognosis of lung cancer patients over the past few decades, the 5-year survival rate for lung cancer remains under 20% in most countries [1]. Therefore, there is an urgent need to develop novel strategies and therapeutics for the treatment of lung cancer.

Epidermal growth factor receptor (EGFR) is an important target for lung cancer therapy because EGFR mutations are frequently observed in lung cancer [2]. These EGFR-activating mutations contribute to dramatic responses to first-generation EGFR tyrosine kinase inhibitors (TKIs), including erlotinib and gefitinib [3]. However, acquired resistance to EGFR TKIs emerges within a year, primarily due to the secondary EGFR mutation (T790M) and the activation of alternative pathways, including the MET pathway [4]. Despite the development of next-generation EGFR TKIs to overcome EGFR TKI resistance, the emergence of additional resistance to these drugs has been reported [5]. Hence, more potent strategies to augment the efficacy of EGFR TKIs are needed.

Angiogenesis is a process by which existing endothelial cells of blood vessels migrate and sprout to form vascular structures, driven by various factors including vascular endothelial growth factor (VEGF) and fibroblast growth factor (FGF) [6]. Although angiogenesis is largely inhibited in adults, except in specific circumstances, such as wound healing, it becomes highly activated during cancer progression [6]. Newly formed vessels within tumors exhibit excessive branching, twisting, and dilation, with fragile vessel walls prone to leakage, resulting in microbleeding and irregular blood flow [7]. Because angiogenesis promotes cancer progression by supplying more nutrients and oxygen to the tumor site and facilitating the intravasation of cancer cells to metastasize to distant organs, blocking angiogenesis has been a promising anticancer strategy [6]. For example, bevacizumab, an antibody against VEGF, and sorafenib, a tyrosine kinase inhibitor targeting VEGF receptor 2 (VEGFR2), have been approved by the FDA as cancer therapeutics [6]. Notably, recent studies have reported that anti-angiogenic drugs enhanced the efficacy of EGFR TKIs, suggesting that targeting angiogenesis can be a novel strategy to overcome EGFR TKI resistance [8,9].

The root of *Adenophora triphylla* var. *japonica* (AT), known as Sa-sam in Korea, has traditionally been used in East Asian countries for the treatment of inflammatory airway diseases. Modern studies have shown that AT extracts exhibit diverse pharmacological activities, including anti-obesity, anti-fungal, and anticancer effects [10,11,12,13,14,15,16]. Saponins isolated from AT roots inhibited cancer cell growth and induced apoptosis and autophagic cell death in cancer cells [12,13]. The ethyl acetate fraction of AT root triggered apoptosis and G1 cell cycle arrest in cancer cells through a p53-dependent mechanism [14]. Our previous studies have also demonstrated that AT extracts exert anticancer activities by eliciting apoptosis in lung cancer cells and inhibiting macrophage polarization into the immunosuppressive M2 phenotype [15,16]. However, the influence of AT extracts on angiogenesis remains unexplored. Therefore, the aim of this study was to investigate the effect of AT extracts on angiogenesis and the crosstalk between endothelial cells (ECs) and cancer cells.

## 2. Results

### 2.1. Investigation of AT Root Fractions with Anti-Angiogenic Effects

**Figure 1:**
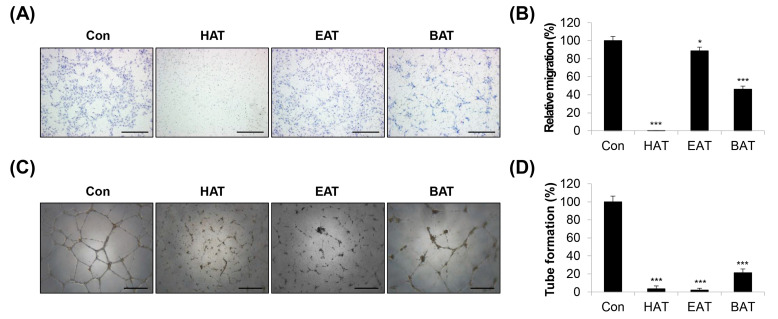
Effects of AT root fractions on migration and tube formation of HUVECs. (**A**) The migratory ability of HUVECs was assessed via a transwell assay after treatment with HAT, EAT, and BAT (50 μg/mL). The migrated cells were photographed (×100 magnification; scale bar = 200 μm). (**B**) Relative migration compared to control cells was determined by counting the stained cells. (**C**) Tube formation of HUVECs after treatment with HAT, EAT, and BAT (50 μg/mL) was photographed (×50 magnification; scale bar = 0.4 μm). (**D**) Quantification of tube formation was conducted by counting the number of loops. Statistical analyses were performed using one-way ANOVA followed by Tukey’s post hoc test. * *p* < 0.05, *** *p* < 0.001 vs. control cells. BAT—butanol fraction of *Adenophora triphylla* var. *japonica* root extract; EAT—ethyl acetate fraction of *Adenophora triphylla* var. *japonica* root extract; HAT—hexane fraction of *Adenophora triphylla* var. *japonica* root extract; HUVEC—human umbilical vein endothelial cell.

Crude ethanolic extract of AT root was subjected to stepwise fractionation using hexane, ethyl acetate, and butanol. The lyophilized powders of the hexane, ethyl acetate, and butanol fractions were designated HAT, EAT, and BAT, respectively. The components of the three fractions were identified via GC/MS analysis (Figure S1 and Table S1). Twenty-two components were identified in HAT, 27 components in EAT, and 16 components in BAT. Of these, three components were consistently present in all three fractions, namely, palmitic acid, 9,12-octadecadienoic acid (Z,Z)- and hexadecanoic acid, and 2-hydroxy-1-(hydroxymethyl) ethyl ester. In addition, eleven components were found to be common between HAT and EAT, including tetradecanoic acid, pentadecanoic acid, octadecanoic acid, erucic acid, docosanoic acid, hexadecanoic acid, ethyl ester, squalene, 9,12-octadecadienoic acid (Z,Z)-, methyl ester, chondrillasterol, lanosta-8,24-dien-3-ol, acetate, (3.beta.)-, and friedelan-3-one. Two components were found to be common between HAT and BAT, including 4H-pyran-4-one, 2,3-dihydro-3,5-dihydroxy-6-methyl-, and 5-hydroxymethyl-2-furaldehyde. A total of 6 unique components were detected exclusively in HAT, 13 in EAT, and 11 in BAT. To investigate the anti-angiogenic potential of the three AT root fractions, we first determined their non-cytotoxic concentration ranges in human umbilical vein endothelial cells (HUVECs). The three fractions showed minimal cytotoxicity (cell viability ≥ 90%) at 50 μg/mL in HUVECs. However, 100 μg/mL of these fractions were generally toxic (cell viability ≤ 80%) to HUVECs (Figure S2). Therefore, in further experiments, the maximum concentration of HAT, EAT, and BAT was set at 50 μg/mL. We next evaluated the anti-angiogenic properties of the AT root fractions using a transwell migration assay and a tube formation assay. To exclude any potential effects of the vehicle, the control group was treated with 0.05% DMSO. Among the three fractions, HAT significantly reduced the migration of HUVECs (Figure 1A,B). The inhibitory effect on tube formation was clearly observed in all three fractions, especially in HAT and EAT (Figure 1C,D). The integration of the two experimental results suggested that HAT had the most pronounced anti-angiogenic effects. Therefore, the following experiments were conducted using HAT. The IC_50_ values for HAT in H1299 and PC9 human lung carcinoma cells were 258.84 ± 15.39 μg/mL and 258.57 ± 17.37 μg/mL, respectively. These values were relatively lower compared to the IC_50_ value in normal WI38 human lung fibroblasts (589.25 ± 11.39 μg/mL), suggesting that cancer cells exhibit greater sensitivity to HAT than normal cells (Figure S3).

### 2.2. Effects of HAT on the Angiogenic Capacity of HUVECs

**Figure 2:**
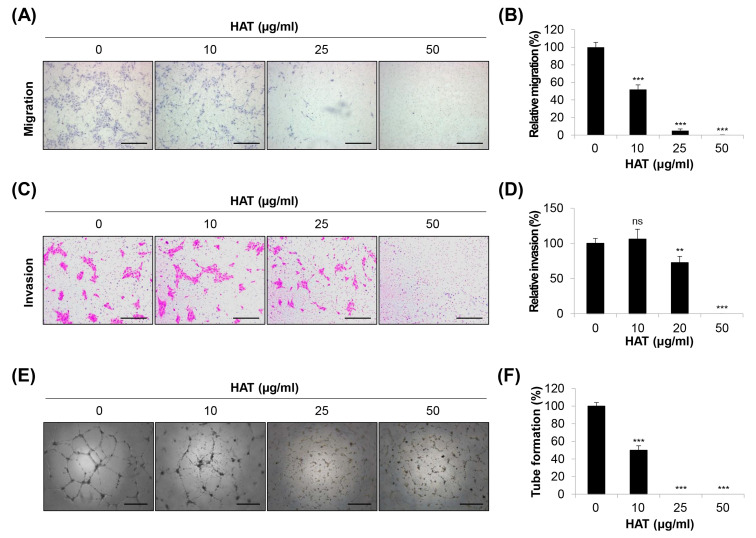
Effects of HAT on the angiogenic capacity of HUVECs. The migratory ability (**A**,**B**) and invasive capacity (**C**,**D**) of HUVECs were assessed via a transwell assay after treatment with HAT. The migrated (**A**) or invaded (**C**) cells were stained and photographed (×100 magnification; scale bar = 200 μm). Relative migration (**B**) and invasion (**D**) compared to control cells were evaluated by counting the stained cells. (**E**) Tube formation of HUVECs after treatment with HAT was photographed (×50 magnification; scale bar = 0.4 μm). (**F**) Quantification of tube formation was conducted by counting the number of loops. Statistical analyses were performed using one-way ANOVA followed by Tukey’s post hoc test. ns—not significant, ** *p* < 0.01, *** *p* < 0.001 vs. control cells. HAT—hexane fraction of *Adenophora triphylla* var. *japonica* root extract; HUVEC—human umbilical vein endothelial cell; VEGF—vascular endothelial growth factor.

To further explore the anti-angiogenic properties of HAT, HUVECs were treated with varying concentrations of HAT (10–50 μg/mL). Our results showed that HAT suppressed the migration of HUVECs by nearly 50% at 10 μg/mL and completely blocked this capability at 25 μg/mL and 50 μg/mL (Figure 2A,B). Similarly, the invasive capacity of HUVECs also exhibited a concentration-dependent decrease upon HAT treatment (Figure 2C,D). This trend was consistently observed in the tube formation assay. Moreover, 25 μg/mL of HAT was sufficient to completely inhibit the tube-forming ability of HUVECs (Figure 2E,F). Taken together, these results suggest that HAT effectively inhibits the angiogenic capacity of HUVECs.

### 2.3. Effects of HAT on the VEGFR2 Signaling Pathway in HUVECs

**Figure 3:**
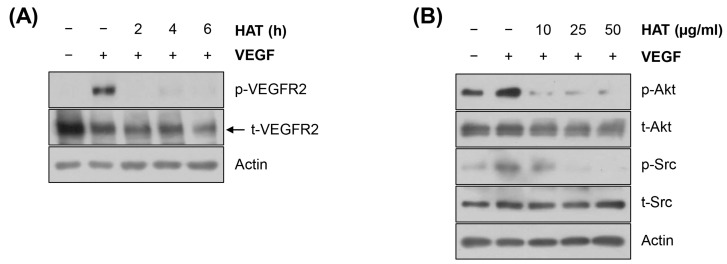
Effects of HAT on the VEGFR2 signaling pathway in HUVECs. The phosphorylation and total protein expression of VEGFR2 (**A**), AKT, and Src (**B**) in HUVECs were assessed via Western blot. Actin was used as an internal control. HAT—hexane fraction of *Adenophora triphylla* var. *japonica* root extract; HUVEC—human umbilical vein endothelial cell; VEGF—vascular endothelial growth factor; VEGFR2—vascular endothelial growth factor receptor 2.

The VEGFR2 signaling pathway is critically involved in tumor angiogenesis. Under hypoxic conditions, cancer cells and ECs produce VEGF and subsequently activate the VEGFR2 signaling pathway, which, in turn, stimulates EC proliferation/survival, migration/invasion, and tube formation [17]. As a general intermediate of the VEGFR2 pathway, the phosphoinositide 3-kinase (PI3K)/AKT axis contributes to EC tube formation, proliferation, and vascular permeability [18]. Src, another downstream component of the VEGFR2 pathway, regulates the cytoskeleton in ECs and vascular endothelial (VE) cadherin in adherent junctions to enhance EC migration and permeability [19,20]. Based on the critical role of the VEGFR2 signaling pathway in tumor angiogenesis, we next investigated the effects of HAT on regulating the VEGFR2 pathway in HUVECs. As shown in Figure 3A, VEGF significantly increased the phosphorylation level of VEGFR2, which was completely abrogated by HAT treatment. In addition, VEGF-stimulated phosphorylation of AKT and Src was consistently suppressed by HAT in a concentration-dependent manner (Figure 3B). Taken together, these results suggest that HAT inhibits the VEGFR2 signaling pathway in HUVECs.

### 2.4. Binding of HAT Constituents to VEGFR2 Kinase Domain

**Figure 4:**
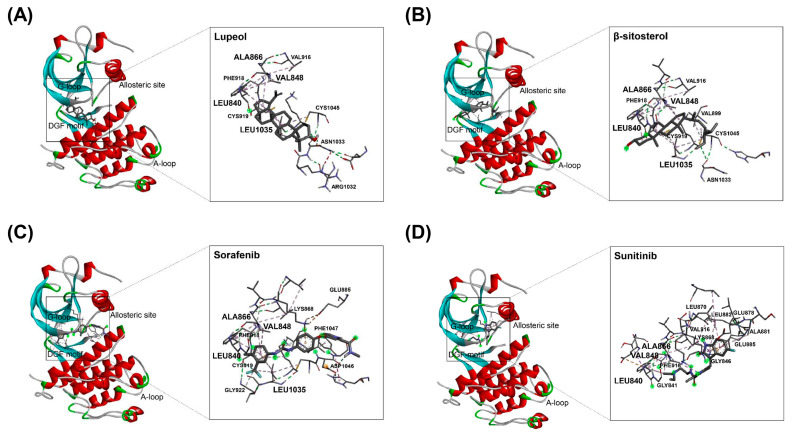
Binding modes of HAT constituents and VEGFR2 inhibitors to VEGFR2 kinase domain. Docking analysis was performed to investigate the structural interactions between VEGFR2 kinase domain and various compounds: (**A**) lupeol, (**B**) β-sitosterol, (**C**) sorafenib, and (**D**) sunitinib. HAT—hexane fraction of *Adenophora triphylla var. japonica* root extract; VEGFR2—vascular endothelial growth factor receptor 2.

In our previous study, GC-MS fingerprints demonstrated that lupeol and β-sitosterol, representative components of AT roots, were contained in HAT [15]. To further investigate whether the inhibitory effect of HAT on VEGFR2 activity is related to the functions of lupeol and β-sitosterol, we conducted molecular docking analysis. We utilized the crystal structure of VEGFR2 kinase domain (PDB ID: 1VR2) for this analysis. Sorafenib and sunitinib, both inhibitors of VEGFR2, were used as controls. Among various binding modes between lupeol and VEGFR2, and between β-sitosterol and VEGFR2, the 3D binding modes with the lowest binding ΔG were shown (Figure 4A,B). Our results clearly revealed that lupeol and β-sitosterol bind to the VEGFR2 kinase domain with binding ΔG values of −8.5 and −7.9, respectively (Figure 4A,B and Table 1). These values were comparable to that of sorafenib (−8.0) and even superior to that of sunitinib (−7.2) (Figure 4C,D and Table 1). Thus, our findings suggest that lupeol and β-sitosterol have the potential to inhibit VEGFR2 activity by directly binding to VEGFR2, which may contribute to the inhibitory effect of HAT on the VEGFR2 signaling pathway.

### 2.5. Effects of HAT on Cancer-Cell-Induced Chemotaxis of HUVECs

**Figure 5:**
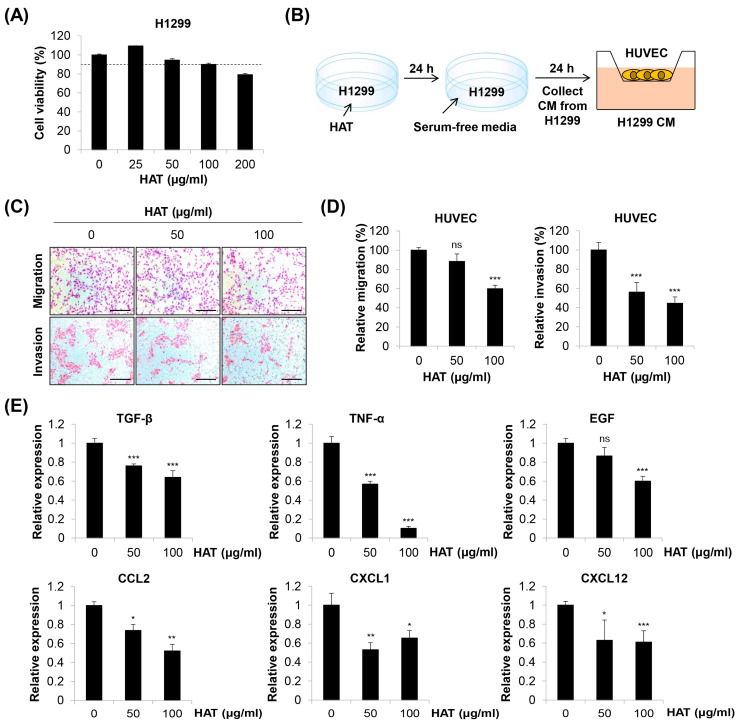
Effects of HAT on cancer cell-induced chemotaxis of HUVECs. (**A**) Effect of HAT on the cell viability of H1299 human lung cancer cells was assessed via MTT assay. The dotted line indicates a threshold of 90% cell viability. (**B**) The experimental scheme is shown. (**C**) The migrated (upper panel) or invaded (lower panel) HUVECs were photographed (×100 magnification; scale bar = 200 μm). (**D**) Relative migration (left panel) and invasion (right panel) compared to control cells were evaluated by counting the stained cells. (**E**) The mRNA expression of the indicated genes in H1299 cells was measured via real-time PCR. Statistical analyses were performed by one-way ANOVA followed by Tukey’s post hoc test. ns, not significant, * *p* < 0.05, ** *p* < 0.01, *** *p* < 0.001 vs. control cells. HAT—hexane fraction of *Adenophora triphylla* var. *japonica* root extract; HUVEC—human umbilical vein endothelial cell; CM—conditioned medium; TGF-β—transforming growth factor-β; TNF-α—tumor necrosis factor-α; EGF—epidermal growth factor; CCL2—C-C motif chemokine ligand 2; CXCL1—C-X-C motif chemokine ligand 1.

Cancer cells produce numerous factors to attract ECs and stimulate angiogenesis. ECs express various receptors that bind to these ligands [21]. To investigate the influence of HAT on cancer-cell-induced EC chemotaxis, we collected conditioned medium (CM) from HAT-pretreated H1299 human lung cancer cells. The maximum concentration of HAT was set at 100 μg/mL for H1299 cells based on the threshold of cell viability ≥ 90% (Figure 5A). HUVECs suspended in basal medium without growth factors were seeded in the upper chambers, and the HAT-pretreated H1299 CM was added to the lower chambers as a chemoattractant. The chemotactic effect of H1299 CM on HUVECs was assessed by quantifying the number of migrated or invaded cells (Figure 5B). As shown in Figure 5C,D, the pretreatment of H1299 cells with HAT attenuated the migration and invasion of HUVECs. Among various angiogenic or chemotactic factors for ECs, HAT consistently down-regulated the expressions of transforming growth factor (TGF)-β, tumor necrosis factor (TNF)-α, epidermal growth factor (EGF), C-C motif chemokine ligand (CCL) 2, C-X-C motif chemokine ligand (CXCL) 1, and CXCL12 in H1299 cells (Figure 5E). Taken together, these results suggest that HAT inhibits cancer-cell-induced EC chemotaxis by decreasing the production of multiple angiogenic and chemotactic factors by cancer cells.

### 2.6. Effects of HAT on EC-Induced EGFR TKI Resistance in Cancer Cells

**Figure 6:**
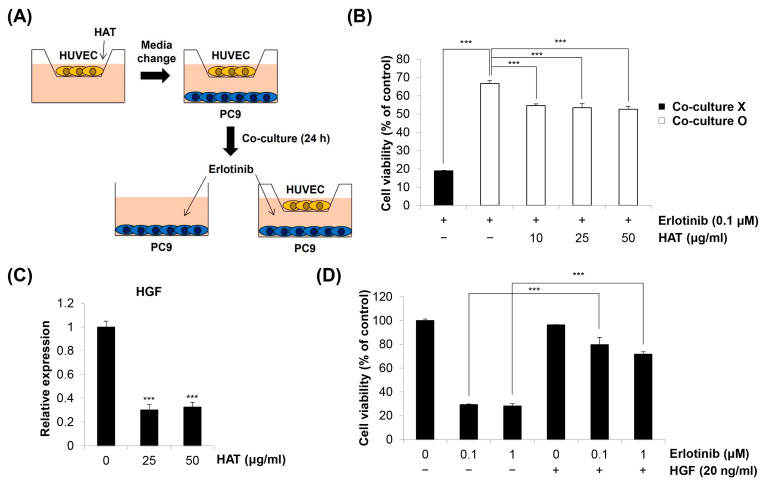
Effects of HAT on EC-induced EGFR TKI resistance in cancer cells. (**A**) The experimental scheme is shown. (**B**) The cell viability of PC9 cells monocultured or co-cultured with HUVECs was assessed via MTT assay following erlotinib treatment. (**C**) The mRNA expression of HGF in HUVECs treated with HAT was measured via real-time PCR. (**D**) PC9 cells were treated with erlotinib with or without HGF for 72 h. Cell viability was assessed via MTT assay. Statistical analyses were performed using one-way ANOVA followed by Tukey’s post hoc test. *** *p* < 0.001 vs. respective control. HAT—hexane fraction of *Adenophora triphylla* var. *japonica* root extract; EC—endothelial cell; HUVEC—human umbilical vein endothelial cell; HGF—hepatocyte growth factor.

**Figure 7:**
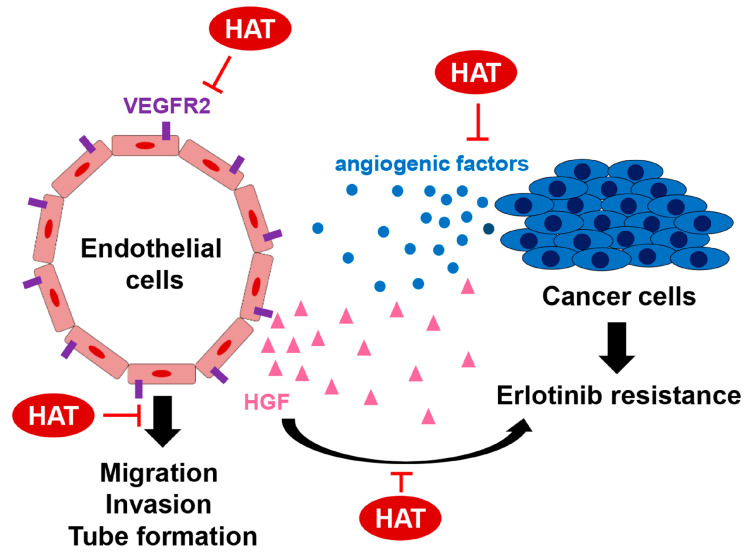
Multifaceted action of HAT on the complex crosstalk between ECs and cancer cells. HAT suppressed the antigenic potential of HUVECs by targeting the VEGFR2 signaling pathway. HAT reduced the production of angiogenic and chemotactic factors by cancer cells. HAT inhibited the development of erlotinib resistance in PC9 cells by attenuating HGF production from ECs.

As HAT suppressed the angiogenic properties of HUVECs and cancer-cell-induced HUVEC chemotaxis, we next investigated how these anti-angiogenic effects of HAT influence cancer cell behavior. The role of ECs in cancer progression has been extensively studied [6]. In addition to providing oxygen and nutrients to the tumor site, ECs increase cancer cell motility and invasiveness and promote EMT [22,23]. More recently, targeting angiogenesis has been considered an effective strategy to overcome EGFR TKI resistance [8,9]. Based on these facts, we investigated whether co-culture with HUVECs affects EGFR TKI resistance in cancer cells and whether HAT can counteract this process (Figure 6A). As shown in Figure 6B, erlotinib exhibited potent cytotoxicity in EGFR TKI-sensitive PC9 human lung cancer cells, which was diminished when PC9 cells were co-cultured with HUVECs. Interestingly, co-culture with HAT-pretreated HUVECs partially restored the sensitivity of PC9 cells to erlotinib (Figure 6B). These findings suggest that HAT regulates the HUVEC secretome that confers erlotinib resistance in PC9 cells. We then postulated which factor present in the HUVEC CM is involved in erlotinib resistance. We focused on hepatocyte growth factor (HGF), given that HGF plays a pivotal role in the development of EGFR TKI resistance and can be produced by ECs [24,25]. Our results clearly showed that HAT reduced the expression of HGF in HUVECs (Figure 6C). To confirm that HGF is a key factor regulating erlotinib resistance, we treated PC9 cells with HGF in combination with erlotinib. As previously reported [24], the combined treatment of HGF and erlotinib led to a significant induction of erlotinib resistance in PC9 cells (Figure 6D). Taken together, our results suggest that HAT inhibits the development of erlotinib resistance in PC9 cells by attenuating HGF production from ECs, highlighting the multifaceted action of HAT on the complex crosstalk between ECs and cancer cells (Figure 7).

## 3. Discussion

In this study, we investigated the anticancer effects of HAT, specifically focusing on its anti-angiogenic potential. Our results showed that HAT suppressed the migration, invasion, and tube formation of HUVECs by targeting the VEGFR2 signaling pathway. Furthermore, HAT reduced the production of angiogenic and chemotactic factors by cancer cells. The erlotinib resistance in PC9 cells induced by co-culture with HUVECs was attenuated by the pretreatment of HUVECs with HAT, which was due to the decrease in HGF secretion from HUVECs. Therefore, the novelties of this study are as follows: (i) this is the first study to report the anti-angiogenic effects of HAT and elucidate its underlying mechanism, and (ii) this study focused on the multifaceted action of HAT on the complex crosstalk between ECs and cancer cells to regulate EGFR TKI resistance.

The superior anti-angiogenic activity of HAT compared to EAT or BAT could be due to the different composition of the three fractions, as demonstrated by the results of GC/MS analysis. Among twenty-two components identified in HAT, three components were consistently present in all three fractions, namely, palmitic acid, 9,12-octadecadienoic acid (Z,Z)- and hexadecanoic acid, 2-hydroxy-1-(hydroxymethyl) ethyl ester. When considering the peak areas of the total chromatogram as relative abundance ratios, palmitic acid and 9,12-octadecadienoic acid (Z,Z) were found to be the most abundant components in both HAT and EAT. Notably, palmitic acid was reported to inhibit endothelial cell proliferation, migration, and tube formation [26]. However, considering that the anti-angiogenic activity of HAT was superior to that of EAT, and the abundance ratios of these compounds did not differ significantly between HAT and EAT, it is challenging to attribute the effects of HAT solely to these components. Moreover, the 11 components present in both HAT and EAT did not exhibit significant differences in abundance ratios between the two fractions, making it difficult to consider them as active compounds. The compounds 4H-Pyran-4-one, 2,3-dihydro-3,5-dihydroxy-6-methyl, and 5-Hydroxymethyl-2-furaldehyde, which were common to both HAT and BAT, were excluded from consideration as active components due to their relatively higher abundance in BAT. Consequently, six components exclusively present in HAT may be considered potential active compounds. Among them, 5-hydroxymethyl-2-furaldehyde, D-allose, asarone, and α-tocopherol were reported to have anti-cancer activities, although their anti-angiogenic effects have not been elucidated [27,28,29,30]. Whether these compounds possess anti-angiogenic effects and can target VEGFR2 should be elucidated through future studies. It is important to note, however, that not all peaks in the total chromatograms of HAT, EAT, and BAT were identified. Therefore, such interpretations should be validated through more detailed investigations in the future. This will contribute to a better understanding of the mechanisms underlying the anti-angiogenic effects of HAT.

From the perspective of cancer cells, HAT down-regulated the expression of TGF-β, TNF-α, EGF, CCL2, CXCL1, and CXCL12, crucial angiogenic and chemotactic factors for ECs. Among them, both TGF-β and TNF-α have been reported to promote EC migration by inducing the endothelial-to-mesenchymal transition (EndMT) [31,32,33]. EGF is also known to stimulate HUVEC migration and tube formation by activating the PI3K and MAPK signaling pathways [34]. In addition, CCL2, CXCL1, and CXCL12 have been reported to induce EC chemotaxis [21]. Thus, the reduced expression of these factors by HAT suggests that the recruitment of ECs to a tumor site and the angiogenic properties of ECs can be inhibited by HAT. The inhibition of angiogenesis around the tumor will inhibit cancer growth and metastasis as the cancer cannot receive sufficient oxygen and nutrients. From the perspective of ECs, HAT suppressed the motility, invasiveness, and tube-forming ability of HUVECs and reduced the expression of HGF in HUVECs. HGF is a ligand for MET, a transmembrane tyrosine kinase receptor overactivated in most EGFR TKI-resistant tumors. *MET* amplification is responsible for 5–20% of EGFR TKI resistance [4,24]. Once activated by its ligand, MET promotes the cell proliferation, survival, and metastatic ability of cancer cells [24]. HGF itself is also highly expressed in EGFR TKI-resistant lung cancer [35,36]. Therefore, targeting the HGF/MET axis in EGFR TKI-resistant lung cancer has emerged as a promising strategy to overcome EGFR TKI resistance [35,37]. Our results showed that PC9 cells co-cultured with HUVECs were more resistant to erlotinib than PC9 cells cultured alone. Furthermore, co-culture with HAT-pretreated HUVECs partially restored the sensitivity of PC9 cells to erlotinib. We propose that HUVEC-derived HGF is a key factor in regulating erlotinib resistance, based on the following results: (i) HAT reduced the expression of HGF in PC9 cells, and (ii) the addition of HGF to PC9 cells significantly induced erlotinib resistance. However, the possibility remains that other secreted factors may cooperate with HGF or even play a more important role than HGF in contributing to erlotinib resistance. Further investigations are needed to elucidate the precise mechanism by which HAT counteracts erlotinib resistance induced by HUVECs. Taken together, our results suggest that HAT inhibits angiogenesis and the emergence of drug resistance by regulating the complex crosstalk between cancer cells and ECs.

Despite the novelty of this study, there are several limitations that warrant further investigation. Firstly, the specific compound in HAT that exerts anti-angiogenic effects should be determined. Although our molecular docking analysis results suggest that lupeol and β-sitosterol can directly bind to the kinase domain of VEGFR2, validation is required to confirm whether they indeed inhibit the VEGFR2 signaling pathway. Notably, Das et al. [38] reported a high binding affinity of β-sitosterol for VEGFR2 through an in silico docking approach. Similarly, Qian et al. [39] demonstrated that β-sitosterol exerts anti-angiogenic effects by binding to VEGFR2 and suppressing its phosphorylation. Furthermore, it has been reported that lupeol suppresses angiogenesis in HUVECs by inhibiting the VEGFR2 signaling pathway [40]. These studies consistently suggest that β-sitosterol and lupeol may contribute to the anti-angiogenic effects of HAT. Secondly, the molecular mechanism by which HAT regulates the expression of multiple angiogenic factors in cancer cells should be elucidated. A comprehensive investigation of the secretome of cancer cells and ECs after HAT treatment will provide a broader view of how HAT regulates the complex crosstalk between ECs and cancer cells. Thirdly, in vivo experiments are warranted to validate the anti-angiogenic effects of HAT. Conducting in vivo studies will provide valuable insights into the therapeutic potential of HAT as an anticancer agent. Maintaining consistent quality control for the origin and composition of active compounds and other characteristics of the AT root can be a challenge for the clinical use of HAT.

In conclusion, this study has provided valuable insights into the anti-angiogenic effects of HAT in HUVECs and its influence on EGFR TKI resistance in lung cancer cells. HAT suppressed the migration, invasion, and tube formation of HUVECs and subsequently counteracted EC-induced erlotinib resistance in PC9 cells. The bidirectional effects of HAT on ECs and cancer cells highlight its multifaceted action as a herbal medicine. Further preclinical and clinical investigations are warranted to develop HAT as a novel therapeutic agent for lung cancer patients.

## 4. Materials and Methods

### 4.1. Preparation of HAT

Dried roots of AT (batch number 066500180) originating from Dongbei, China, were purchased from Nuri Herb Co., Ltd. (Yeongcheon, Republic of Korea). Authentication of the sample, including sensory evaluation and identification tests, was carried out by Shinhung Pharmaceutical Co., Ltd. (Yeosu, Republic of Korea). A voucher specimen is deposited in the herbarium of Pathology Laboratory, College of Korean Medicine, Dong-eui University, Busan, Republic of Korea. The methodology for preparing HAT was described in detail in our previous study [16]. The AT roots (500 g) were pulverized and extracted with 4 L of 80% ethanol in a shaking incubator (100 rpm) for 24 h at room temperature. The crude ethanolic extract of AT roots was lyophilized, dissolved in 500 mL distilled water (DW), and subjected to stepwise fractionation using hexane, ethyl acetate, and butanol. Initially, hexane was added in a 1:1 ratio, and the upper hexane layer was separated after shaking in a separatory funnel. Ethyl acetate was added to the remaining layer after the hexane separation in the same volume, and the ethyl acetate layer was obtained using the same procedure as described above. Subsequently, butanol was added in a 1:1 ratio to the remaining layer after separation of the ethyl acetate layer, and the butanol layer was obtained using a similar procedure. The lyophilized powders of the hexane, ethyl acetate, and butanol layers were designated as HAT, EAT, and BAT, respectively. They were dissolved in dimethyl sulfoxide (DMSO; Amresco, Solon, OH, USA) as a stock solution. The gas chromatography (GC-MS) fingerprints demonstrated that β-sitosterol and lupeol, representative components of AT roots, were contained in HAT [15].

### 4.2. Cell Culture

Human umbilical vein endothelial cells (HUVECs) were purchased from Lifeline Cell Technology (Frederick, MD, USA). H1299 human lung cancer cell line was purchased from American Type Culture Collection (Rockville, MD, USA). PC9 human lung cell line was kindly provided by Professor Ho-Young Lee (Seoul National University, Seoul, Republic of Korea). HUVECs were cultured in VascuLife^®^ VEGF Endothelial Medium supplemented with growth factors (VascuLife^®^ VEGF Endothelial Medium Complete Kit, Lifeline Cell Technology). This medium is referred to as “growth medium”. H1299 and PC9 cells were cultured in RPMI-1640 medium (WelGENE, Daegu, Republic of Korea) supplemented with 10% fetal bovine serum (FBS; WelGENE) and 1% antibiotics (WelGENE). All cells were maintained at 37 °C under 5% CO_2_ conditions.

### 4.3. MTT Assay

Cells were seeded in 96-well culture plates at a density of 1 × 10^4^ cells/well (for HUVEC) or 4 × 10^3^ cells/well (for H1299). Following overnight stabilization, the cells were exposed to varying concentrations of HAT, EAT, and BAT (10–200 μg/mL) for 24 h. PC9 cells were seeded in 96-well culture plates at a density of 3 × 10^3^ cells/well and treated with erlotinib (0.1 μM or 1 μM) with or without HGF (20 ng/mL) for 72 h. Subsequently, a solution of 3-(4,5-dimethylthiazol-2-yl)-2,5-diphenyltetrazolium bromide (MTT; Duchefa, Haarlem, The Netherlands) was added to each well at 0.4 mg/mL. After 2 h of incubation at 37 °C, the culture medium was removed, and 100 μL of DMSO was added to dissolve the MTT formazan crystals. The cell viability was quantified by measuring the absorbance of the formazan solution at 540 nm using a microplate reader (SpectraMax M3; Molecular Devices, San Jose, CA, USA).

### 4.4. Transwell Assay

For transwell migration assay, the outer membranes of transwell inserts with a pore size of 8.0 μm (Corning Inc., Corning, NY, USA) in 24-well format were coated with 0.1% gelatin (Sciencell, Carlsbad, CA, USA). HUVECs (5 × 10^4^ cells/insert) suspended in basal medium (without growth factors) were seeded onto the inserts and treated with HAT, EAT, and BAT (50 μg/mL) or varying concentrations of HAT (10–50 μg/mL). Conditioned medium obtained from H1299 cells (H1299 CM) was added to the lower chamber as a chemoattractant. After 24 h, the migrated cells were fixed in methanol, stained with hematoxylin (Sigma-Aldrich, St Louis, MO, USA), and thoroughly rinsed with DW. After gently wiping the inner surface of the inserts with a cotton swab, the stained cells were photographed at ×100 magnification and counted under a microscope (Carl Zeiss, Oberkochen, Germany). The transwell invasion assay was performed using the same protocol as the transwell migration assay, except that the inner surface of the inserts was pre-coated with 300 μg/mL Matrigel (BD Bioscience, San Jose, CA, USA). The H1299 CM was prepared as follows: H1299 cells untreated or pretreated with HAT (50–100 μg/mL) for 24 h were allowed to reach confluence. The culture medium was then replaced with serum-free RPMI-1640 medium. Following an additional 24 h of incubation, the culture medium was collected and used for experiments.

### 4.5. Tube Formation Assay

A 96-well culture plate was coated with 50 μL of growth factor-reduced Matrigel (Corning Inc.). HUVECs suspended in growth medium were then seeded in the 96-well culture plate at a density of 2 × 10^4^ cells/well and treated with HAT, EAT, and BAT (50 μg/mL) or different concentrations of HAT (10–50 μg/mL). After 24 h of incubation at 37 °C, tube formation was observed under a microscope (Carl Zeiss) at ×50 magnification.

### 4.6. Reverse Transcription Quantitative Polymerase Chain Reaction (RT-qPCR)

H1299 cells were treated with HAT (50–100 μg/mL) for 24 h. HUVECs were treated with HAT (25–50 μg/mL) for 24 h. Total RNA was extracted using TRIzol reagent (Thermo Fisher Scientific, San Jose, CA, USA) and was quantified using a microplate reader (Molecular Devices). First-strand cDNA was synthesized using CellScript™ cDNA Master Mix (CellSafe, Suwon, Republic of Korea). The cDNA templates were diluted 10-fold in nuclease-free water and subjected to qPCR analysis using TOPreal™ SYBR Green qPCR PreMIX (Enzynomics, Daejeon, Republic of Korea) on a CFX Connect Real-Time PCR Detection System (Bio-Rad Laboratories, Hercules, CA, USA). The specific primer sequences and annealing temperatures for each target gene are shown in Table 2.

### 4.7. Western Blot

HUVECs were treated with HAT (50 μg/mL) for different time periods (2–6 h) or treated with different concentrations of HAT (10–50 μg/mL) for 12 h and then stimulated with VEGF (20 ng/mL) 10 min before harvest. Detailed procedures of Western blot analysis were described in our previous study [41]. Briefly, total proteins were extracted using RIPA buffer (Thermo Fisher Scientific). A total of 20 μg protein for each sample was separated using sodium dodecyl sulfate (SDS)–polyacrylamide gel electrophoresis (PAGE) and transferred onto polyvinylidene fluoride (PVDF) membranes. After blocking with 3% bovine serum albumin (BSA, GenDEPOT, Barker, TX, USA), the membranes were probed with corresponding primary antibodies (1:1000 dilution, 4 °C, overnight) and then secondary antibodies (1:5000 dilution, room temperature, 1 h). Specific signals on the membrane were detected using a D-Plus ECL Femto System (Donginbio, Seoul, Republic of Korea). The ratio of phosphorylated/total protein was calculated using ImageJ software (version 1.52a; National Institutes of Health, Bethesda, MD, USA) after normalization to actin. Primary antibodies against phospho-VEGFR2 (Y1175, #2478T), phospho-Src (Y416, #2101S), phospho-AKT (S473, #9271S) were purchased from Cell Signaling Technology (Beverly, MA, USA). Primary antibodies against VEGFR2 (#sc-7269), Src (#sc-8056), AKT (#sc-5298), and actin (#sc-47778) were purchased from Santa Cruz Biotechnology (Santa Cruz, CA, USA). The anti-mouse and anti-rabbit secondary antibodies were purchased from Enzo Life Sciences (Farmingdale, NY, USA).

### 4.8. Co-Culture

HUVECs suspended in growth medium (2 × 10^4^ cells/insert) were added to the inserts of 24-well transwell plates with a pore size of 0.4 μm (Corning Inc.) and treated with HAT (10–50 μg/mL). After 24 h, the culture media of HUVECs were replaced with fresh growth media without HAT to exclude the possibility of direct effects of HAT on PC9 cells. PC9 cells (2 × 10^4^ cells) were then seeded in the lower chambers of 24-well transwell plates for co-culture with HUVECs. After 24 h of co-culture, PC9 cells in the lower chambers were treated with erlotinib (0.1 μM) for 72 h in continuous co-culture with HUVECs. The viability of PC9 cells was assessed via MTT assay.

### 4.9. Molecular Docking Analysis

PyRx - Python Prescription 0.8 was used for the molecular docking analysis. The crystal structure of VEGFR2 kinase domain was obtained from RCSB PDB (ID: 1VR2). The 3D SDF files for lupeol, β-sitosterol, sorafenib, and sunitinib were obtained from PubChem and converted to PDB format using OpenBabel software (version 2.3.1). The binding modes of lupeol, β-sitosterol, sorafenib, and sunitinib to VEGFR2 were visualized using Discovery Studio Visualizer (version 21.1.0.20298).

### 4.10. Gas Chromatography–Mass Spectrometry (GC/MS)

To identify the constituents of HAT, EAT, and BAT, GC-MS analysis was performed using a GCMS-QP2020NX (Shimadzu, Kyoto, Japan). Dissolving HAT, EAT, and BAT in ethanol at a concentration of 20 mg/mL, we prepared aliquots (split ratio of 50:1) of 1 μL, which were then injected into a DB-5MS Ultra column (30 m × 0.25 mm, 0.25 μm). Helium gas served as the carrier gas, maintaining a constant flow rate of 1.0 mL/min. The injection temperature was set at 280 °C, and the ion source temperature was maintained at 200 °C. Electron ionization mode with an electron energy of 70 eV was employed. The oven temperature was programmed to increase from 60 °C (held for 2 min) to 200 °C at 10 °C/min, followed by a further increase to 320 °C at 5 °C/min, where it was held for 20 min. The scan interval was set at 0.3 s, and the fragment size ranged from 40 to 600 *m*/*z*. The entire GC detection process was completed within 60 min.

### 4.11. Statistical Analyses

Each result was presented as the mean ± standard deviation (SD) of data obtained from triplicate experiments. Statistical analyses were performed using one-way ANOVA followed by Tukey’s post hoc test to determine significant differences between groups. Differences with values of *p* < 0.05 were considered statistically significant. Statistical analysis was performed using IBM SPSS Statistics 26.0 (IBM, Chicago, IL, USA).

## Figures and Tables

**Table 1:** Docking of β-sitosterol, lupeol, sorafenib, and sunitinib to VEGFR2 ^1^ kinase domain.

Compound	Binding Residues in Kinase Domain	Binding ΔG
Lupeol	ALA866, VAL848, LEU840, LEU1035	−8.5
β-sitosterol	ALA866, VAL848, LEU840, LEU1035, CYS1045	−7.9
Sorafenib	ALA866, VAL848, LEU840, LEU1035, LYS868, ASP1046, CYS919, PHE1047	−8.0
Sunitinib	ALA866, VAL848, LEU840, ALA881, GLU885, LEU882, LYS868, GLY846, PHE918	−7.2

^1^ Vascular endothelial growth factor receptor 2.

**Table 2:** Primer sequences and annealing temperatures.

Gene	Sequence (5′→3′)	AT ^1^ (°C)
*TGF-β*	F: CCT GTC TGC ACT ATT CCT TT R: TTA TCA GAG TCC CTG CAT CT	55
*TNF-α*	F: CAC CAC GCT CTT CTG TCT ACT G R: GGG CTA CAG GCT TGT CAC TC	58
*EGF*	F: AAG AAT GGG GGT CAA CCA GT R: TGA AGT TGG TTG CAT TGA CC	50
*HGF*	F: GGG CTG AAA AGA TTG GAT CA R: TTG TAT TGG TGG GTG CTT CA	55
*CCL2*	F: CCC CAG TCA CCT GCT GTT AT R: TGG AAT CCT GAA CCC ACT TC	55
*CXCL1*	F: GAA AGC TTG CCT CAA TCC TG R: CAT TAG GCA CAA TCC AGG TG	55
*CXCL12*	F: ATG AAC GCC AAG GTC GTG R: CTT CGG GTC AAT GCA CAC TT	55
*ACTB* (β-actin)	F: GTC TCC TCT GAC TTC AAC AGC G R: ACC ACC CTG TTG CTG TAG CCA A	55

^1^ Annealing temperature.

## Data Availability

The data presented in this study are available on request from the corresponding author.

## Supplementary Materials

The following supporting information can be downloaded at: https://www.mdpi.com/article/10.3390/molecules29030597/s1, Figure S1: Identification of the constituents in AT root fractions by GC/MS analysis. Figure S2: Effects of AT root fractions on HUVEC viability. Figure S3: The IC50 values for HAT on cancer cells and normal cells. Table S1: Characterization of chemical constituents in HAT, EAT, and BAT by GC/MS analysis.
