# Supplementary material for: Hexane Fraction of Adenophora triphylla var. japonica Root Extract Inhibits Angiogenesis and Endothelial Cell-Induced Erlotinib Resistance in Lung Cancer Cells
Source: Molecules. 2024 Jan 25;29(3):597. doi: 10.3390/molecules29030597 (PMC10856037; doi:10.3390/molecules29030597)
Supplement: Supplementary file 1 [file molecules-29-00597-s001.zip › molecules-2823786-supplementary.pdf]

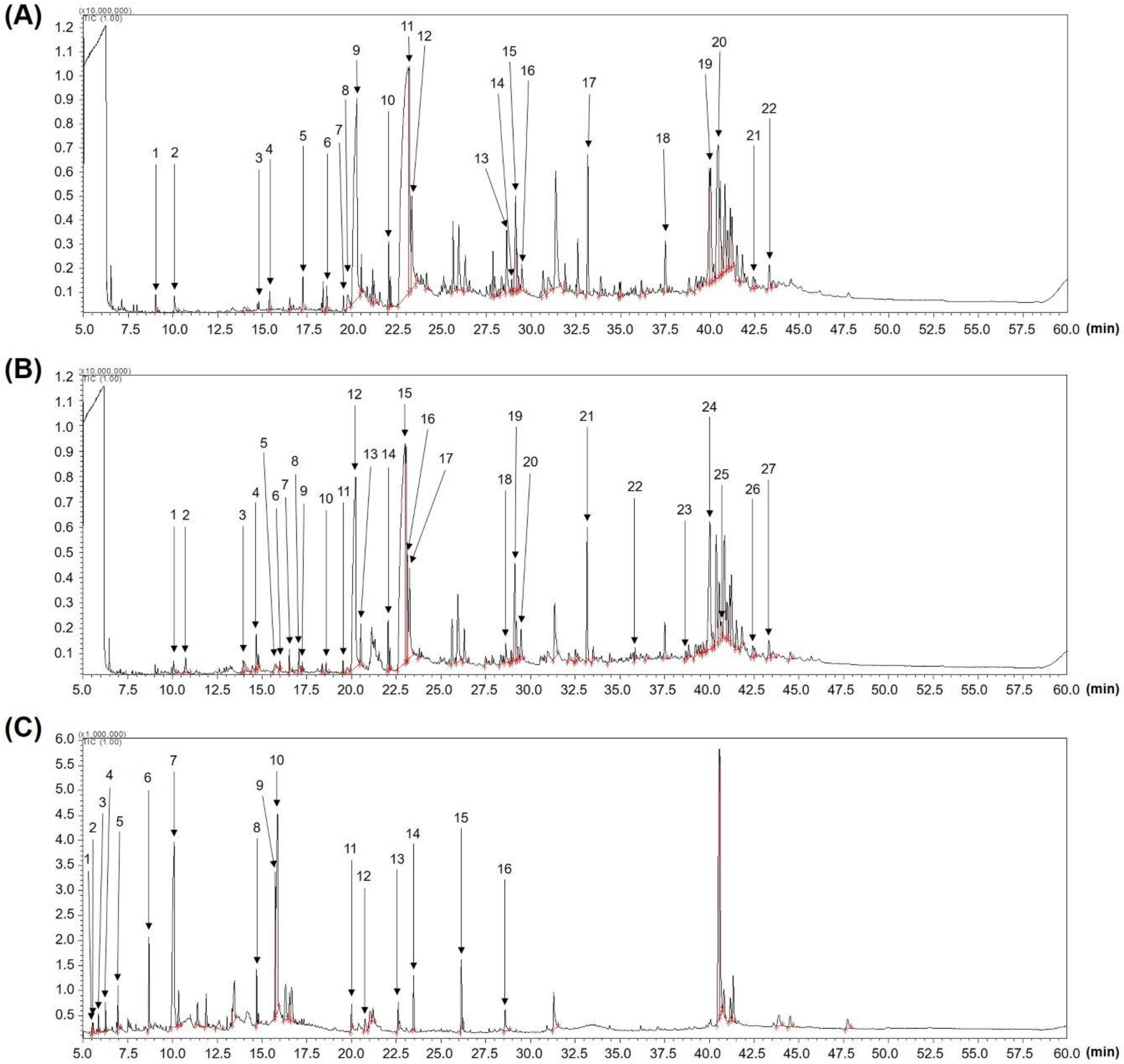

Supplementary Figure S1. Identification of the constituents in AT root fractions by GC/MS analysis. The total gas chromatograms of HAT (A), EAT (B), and BAT (C) are shown. BAT, butanol fraction of *Adenophora triphylla var. japonica* root extract; EAT, ethyl acetate fraction of *Adenophora triphylla var. japonica* root extract; HAT, hexane fraction of *Adenophora triphylla var. japonica* root extract.

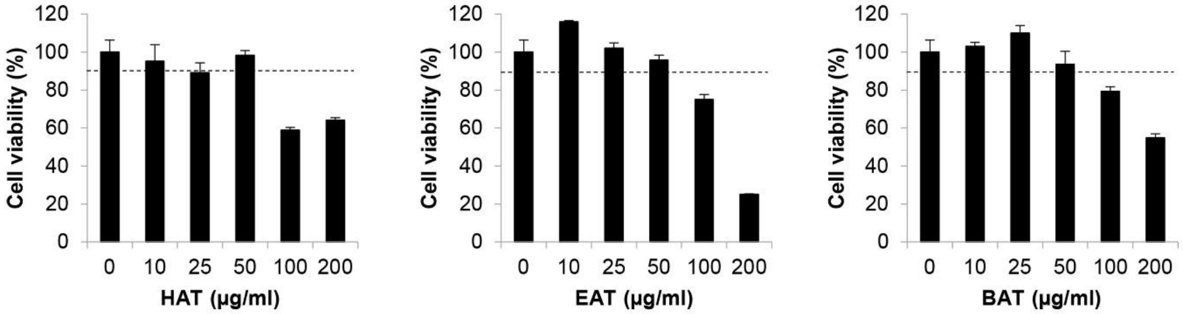

Supplementary Figure S2. Effects of AT root fractions on HUVEC viability. HUVECs were exposed to different concentrations (10-200 μg/ml) of HAT (left panel), EAT (middle panel), and BAT (right panel) for 24 h. Cell viability was measured by MTT assay. The dotted line indicates a threshold of 90% cell viability. BAT, butanol fraction of *Adenophora triphylla var. japonica* root extract; EAT, ethyl acetate fraction of *Adenophora triphylla var. japonica* root extract; HAT, hexane fraction of *Adenophora triphylla var. japonica* root extract; HUVEC, human umbilical vein endothelial cell.

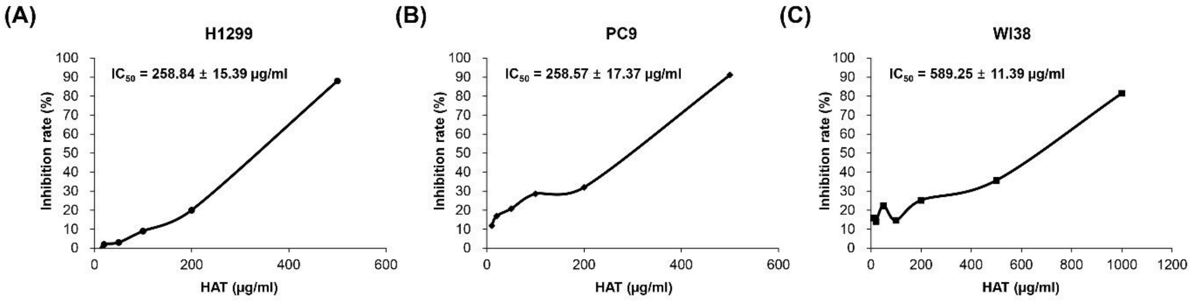

Supplementary Figure S3. The IC_{50} values for HAT on cancer cells and normal cells. H1299 (A) and PC9 (B) human lung carcinoma cells and WI38 human lung fibroblasts (C) were exposed to different concentrations of HAT for 24 h. The growth inhibition rate was determined by assessing cell viability using the MTT assay. The IC_{50} values for HAT were calculated based on the inhibition rate. HAT, hexane fraction of *Adenophora triphylla var. japonica* root extract.

Supplementary Table S1. Characterization of chemical constituents in HAT, EAT, and BAT by GC/MS analysis.

Fraction	No.	RT[1] (min)	Formula	MW[2] (m/z)	SI[3]	Area (%)	Identification
HAT	1	9.037	$C_6H_8O_4$	144	95	0.20	4H-Pyran-4-one, 2,3-dihydro-3,5-dihydroxy-6-methyl-
	2	10.073	$C_6H_6O_3$	126	96	0.36	5-Hydroxymethyl-2-furaldehyde
	3	13.994	$C_6H_{12}O_6$	180	95	0.16	D-Allose
	4	15.400	$C_{12}H_{16}O_3$	208	94	0.17	Asarone
	5	17.258	$C_{14}H_{28}O_2$	228	95	0.44	Tetradecanoic acid
	6	18.594	$C_{15}H_{30}O_2$	242	96	0.35	Pentadecanoic acid
	7	19.769	$C_{16}H_{30}O_2$	254	92	0.53	6-Pentadecenoic acid, 13-methyl-, (6Z)-
	8	20.279	$C_{16}H_{32}O_2$	256	94	12.51	Palmitic acid
	9	20.526	$C_{18}H_{36}O_2$	284	96	0.5	Hexadecanoic acid, ethyl ester
	10	22.051	$C_{19}H_{34}O_2$	294	96	0.83	9,12-Octadecadienoic acid (Z,Z)-, methyl ester
	11	23.139	$C_{18}H_{32}O_2$	280	93	27.33	9,12-Octadecadienoic acid (Z,Z)-
	12	23.348	$C_{18}H_{36}O_2$	284	92	2.29	Octadecanoic acid
	13	28.644	$C_{19}H_{38}O_4$	330	94	1.27	Hexadecanoic acid, 2-hydroxy-1-(hydroxymethyl)ethyl ester
	14	28.926	$C_{24}H_{38}O_4$	390	95	0.21	Diisooctylphthalate
	15	29.146	$C_{22}H_{42}O_2$	338	93	2.22	Erucic acid
	16	29.485	$C_{22}H_{44}O_2$	340	93	0.63	Docosanoic acid
	17	33.176	$C_{30}H_{50}$	410	93	2.65	Squalene
	18	37.502	$C_{29}H_{50}O_2$	430	94	1.06	DL-alpha-Tocopherol

	19	39.960	$C_{30}H_{48}O$	424	90	2.96	Taraxerone
	20	40.034	$C_{29}H_{48}O$	412	94	2.82	Chondrillasterol
	21	42.416	$C_{32}H_{52}O_2$	468	91	0.27	Lanosta-8,24-dien-3-ol, acetate, (3.beta.)-
	22	43.315	$C_{30}H_{50}O$	426	92	0.60	Friedelan-3-one
EAT	1	10.062	$C_6H_6O_3$	126	96	0.28	5-Hydroxymethylfurfural
	2	10.740	$C_4H_6O_5$	134	91	0.43	Malic Acid
	3	14.012	$C_6H_{10}O_5$	162	93	0.39	beta.-D-Glucopyranose, 1,6-anhydro-
	4	14.683	$C_{10}H_{12}O_2$	164	97	0.62	2-Butanone, 4-(4-hydroxyphenyl)-
	5	15.737	$C_9H_{16}O_4$	188	93	0.21	Azelaic acid
	6	16.005	$C_9H_{10}O_4$	182	95	0.12	Benzaldehyde, 4-hydroxy-3,5-dimethoxy-
	7	16.531	$C_{11}H_{14}O_3$	194	93	0.40	(E)-2,6-Dimethoxy-4-(prop-1-en-1-yl)phenol
	8	17.062	$C_{10}H_{12}O_3$	180	91	0.49	(E)-4-(3-Hydroxyprop-1-en-1-yl)-2-methoxyphenol
	9	17.242	$C_{14}H_{28}O_2$	228	94	0.12	Tetradecanoic acid
	10	18.585	$C_{15}H_{30}O_2$	242	96	0.22	Pentadecanoic acid
	11	19.524	$C_{17}H_{34}O_2$	270	96	0.18	Hexadecanoic acid, methyl ester
	12	20.233	$C_{16}H_{32}O_2$	256	94	13.22	Palmitic acid
	13	20.524	$C_{18}H_{36}O_2$	284	95	0.65	Hexadecanoic acid, ethyl ester
	14	22.051	$C_{19}H_{34}O_2$	294	95	0.80	9,12-Octadecadienoic acid (Z,Z)-, methyl ester
	15	23.001	$C_{18}H_{32}O_2$	280	94	24.74	9,12-Octadecadienoic acid (Z,Z)-
	16	23.118	$C_{20}H_{36}O_2$	308	91	1.96	Linoleic acid ethyl ester
	17	23.258	$C_{18}H_{36}O_2$	284	93	3.10	Octadecanoic acid

	18	28.621	$C_{19}H_{38}O_4$	330	94	0.55	Hexadecanoic acid, 2-hydroxy-1-(hydroxymethyl)ethyl ester
	19	29.130	$C_{22}H_{42}O_2$	338	93	2.98	Erucic acid
	20	29.476	$C_{22}H_{44}O_2$	340	93	0.89	Docosanoic acid
	21	33.168	$C_{30}H_{50}$	410	92	3.17	Squalene
	22	35.843	$C_{30}H_{50}O$	426	92	0.24	1,6,10,14,18,22-Tetracosahexaen-3-ol,2,6,10,15,19,23-hexamethyl-,(all-E)-(.+/-.)-
	23	38.671	$C_{20}H_{22}O_6$	358	93	0.19	Pinoresinol
	24	40.021	$C_{29}H_{48}O$	412	92	6.18	Chondrillasterol
	25	40.645	$C_{30}H_{50}O$	426	90	0.50	beta.-Amyrin
	26	42.422	$C_{32}H_{52}O_2$	468	92	0.36	Lanosta-8,24-dien-3-ol, acetate, (3.beta.)-
	27	43.322	$C_{30}H_{50}O$	426	93	0.56	Friedelan-3-one
BAT	1	5.516	$C_6H_6O_2$	110	97	0.27	2-Furancarboxaldehyde, 5-methyl-
	2	5.559	C_7H_6O	106	97	0.32	Benzaldehyde
	3	5.864	$C_6H_8O_4$	144	96	0.38	2,4-Dihydroxy-2,5-dimethyl-3(2H)-furan-3-one
	4	6.260	$C_7H_{14}O_3$	146	96	0.56	Butyl lactate
	5	6.957	C_8H_8O	120	91	1.27	Benzeneacetaldehyde
	6	8.689	$C_6H_8O_4$	144	96	3.02	4H-Pyran-4-one, 2,3-dihydro-3,5-dihydroxy-6-methyl-
	7	10.098	$C_6H_6O_3$	126	95	16.12	5-Hydroxymethyl-2-furaldehyde
	8	14.706	$C_{12}H_{22}O_4$	230	97	1.19	Butanedioic acid, dibutyl ester
	9	15.745	$C_{12}H_{22}O_5$	246	94	4.43	ENT-337
	10	15.874	$C_9H_{15}NO_3$	185	96	13.79	DL-Proline, 5-oxo-, butyl ester

11	20.004	$C_{16}H_{32}O_2$	256	95	0.73	Palmitic acid
12	20.750	$C_{11}H_8N_2$	168	95	0.73	9H-Pyrido[3,4-b]indole
13	22.609	$C_{18}H_{32}O_2$	280	96	1.03	9,12-Octadecadienoic acid (Z,Z)-
14	23.471	$C_{20}H_{40}O_2$	312	93	1.67	Hexadecanoic acid, butyl ester
15	26.136	$C_{22}H_{40}O_2$	336	94	2.38	Butyl 9,12-octadecadienoate
16	28.585	$C_{19}H_{38}O_4$	330	94	1.21	Hexadecanoic acid, 2-hydroxy-1-(hydroxymethyl)ethyl ester

[1]RT, retention time; [2] MW, molecular weight; [3] SI, Similarity index
